# Supplementary material for: Neutralizing mAbs against SFTS Virus Gn Protein Show Strong Therapeutic Effects in an SFTS Animal Model
Source: Viruses. 2022 Jul 28;14(8):1665. doi: 10.3390/v14081665 (PMC9416629; doi:10.3390/v14081665)
Supplement: Supplementary file 1 [file viruses-14-01665-s001.zip › viruses-1818743-supplementary.pptx]

## Slide 1
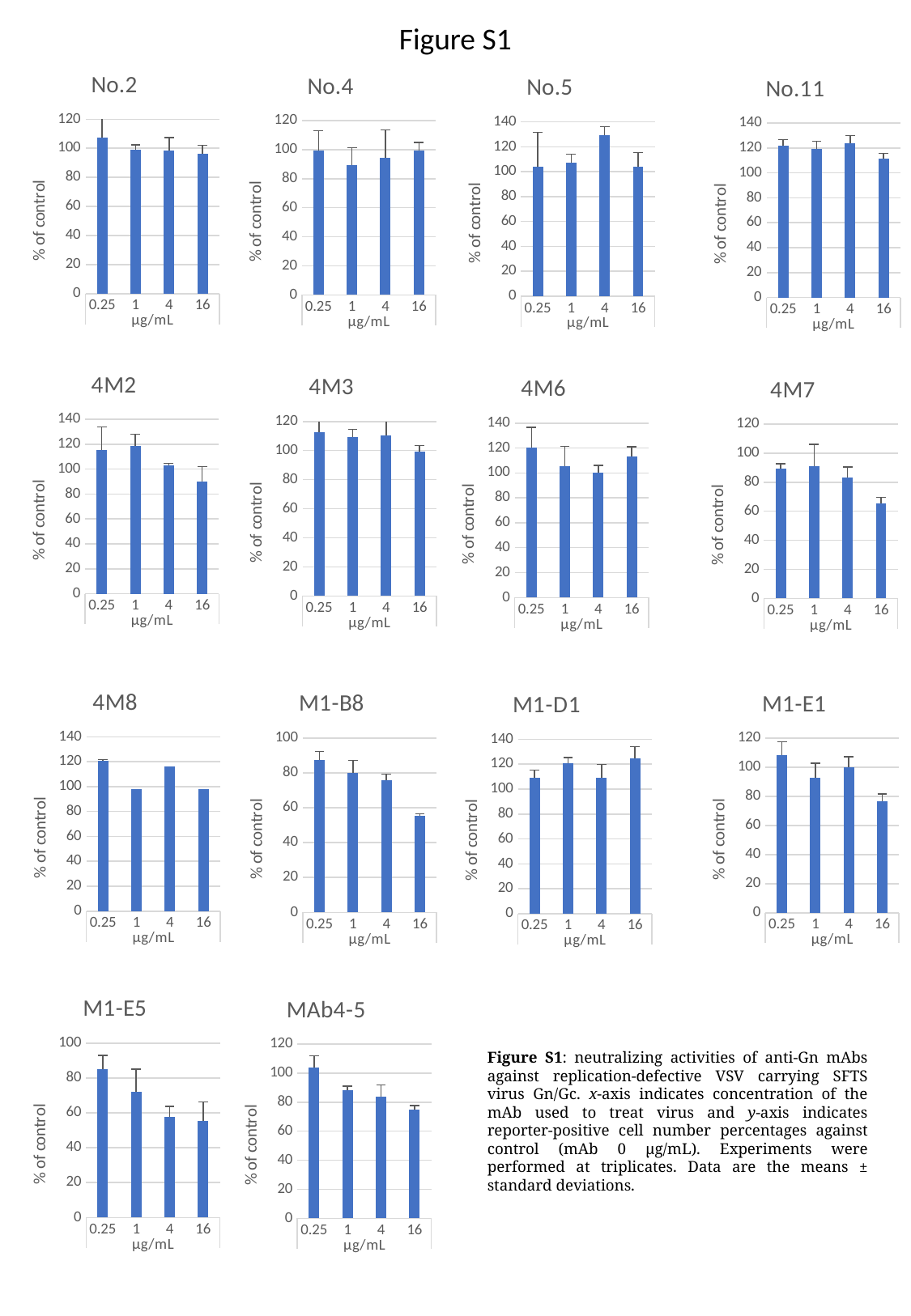

Figure S1
### Chart:
| Category | |
|---|---|
| 0.25 | 107.11678832116787 |
| 1 | 98.9051094890511 |
| 4 | 98.72262773722629 |
| 16 | 96.16788321167884 |
### Chart:
| Category | |
|---|---|
| 0.25 | 99.08759124087594 |
| 1 | 89.05109489051095 |
| 4 | 94.34306569343066 |
| 16 | 99.45255474452556 |
### Chart:
| Category | |
|---|---|
| 0.25 | 103.64963503649635 |
| 1 | 106.93430656934306 |
| 4 | 129.01459854014598 |
| 16 | 103.83211678832119 |
### Chart:
| Category | |
|---|---|
| 0.25 | 121.7153284671533 |
| 1 | 119.16058394160585 |
| 4 | 123.9051094890511 |
| 16 | 111.49635036496352 |
### Chart:
| Category | |
|---|---|
| 0.25 | 115.4639175257732 |
| 1 | 118.76288659793816 |
| 4 | 102.88659793814433 |
| 16 | 90.10309278350515 |
### Chart:
| Category | |
|---|---|
| 0.25 | 112.51700680272108 |
| 1 | 109.52380952380952 |
| 4 | 110.61224489795917 |
| 16 | 99.45578231292518 |
### Chart:
| Category | |
|---|---|
| 0.25 | 120.13605442176872 |
| 1 | 105.44217687074831 |
| 4 | 100.40816326530613 |
| 16 | 112.92517006802721 |
### Chart:
| Category | |
|---|---|
| 0.25 | 89.2464678178964 |
| 1 | 90.97331240188383 |
| 4 | 83.35949764521193 |
| 16 | 65.46310832025118 |
### Chart:
| Category | |
|---|---|
| 0.25 | 120.6436420722135 |
| 1 | 98.03767660910518 |
| 4 | 116.3265306122449 |
| 16 | 97.723704866562 |
### Chart:
| Category | |
|---|---|
| 0.25 | 87.59455370650528 |
| 1 | 80.0302571860817 |
| 4 | 75.642965204236 |
| 16 | 55.37065052950076 |
### Chart:
| Category | |
|---|---|
| 0.25 | 108.16944024205749 |
| 1 | 93.04084720121028 |
| 4 | 100.15128593040846 |
| 16 | 76.85325264750378 |
### Chart:
| Category | |
|---|---|
| 0.25 | 109.06200317965026 |
| 1 | 120.50874403815583 |
| 4 | 109.37996820349763 |
| 16 | 124.48330683624802 |
### Chart:
| Category | |
|---|---|
| 0.25 | 84.8639455782313 |
| 1 | 72.27891156462586 |
| 4 | 57.6530612244898 |
| 16 | 55.27210884353742 |
### Chart:
| Category | |
|---|---|
| 0.25 | 103.91752577319589 |
| 1 | 88.45360824742268 |
| 4 | 83.71134020618557 |
| 16 | 74.63917525773196 |Figure S1: neutralizing activities of anti-Gn mAbs against replication-defective VSV carrying SFTS virus Gn/Gc. x-axis indicates concentration of the mAb used to treat virus and y-axis indicates reporter-positive cell number percentages against control (mAb 0 µg/mL). Experiments were performed at triplicates. Data are the means ± standard deviations.

## Slide 2
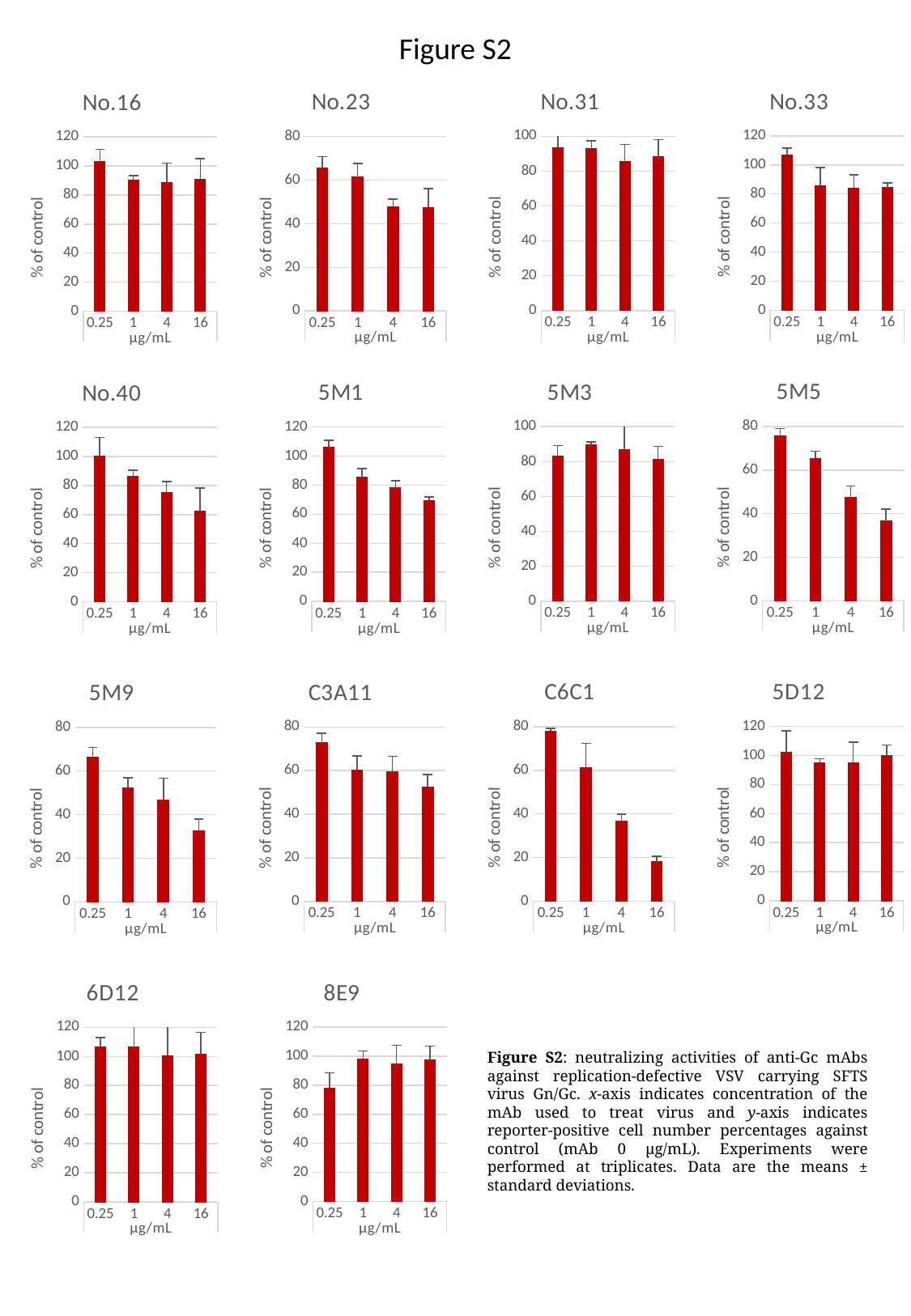

Figure S2
### Chart:
| Category | |
|---|---|
| 0.25 | 107.04419889502763 |
| 1 | 85.91160220994475 |
| 4 | 84.1160220994475 |
| 16 | 84.39226519337018 |
### Chart:
| Category | |
|---|---|
| 0.25 | 93.58059914407988 |
| 1 | 92.7246790299572 |
| 4 | 85.30670470756063 |
| 16 | 88.15977175463622 |
### Chart:
| Category | |
|---|---|
| 0.25 | 65.62054208273894 |
| 1 | 61.62624821683309 |
| 4 | 47.646219686162624 |
| 16 | 47.50356633380884 |
### Chart:
| Category | |
|---|---|
| 0.25 | 103.13837375178316 |
| 1 | 90.01426533523538 |
| 4 | 88.73038516405136 |
| 16 | 91.01283880171184 |
### Chart:
| Category | |
|---|---|
| 0.25 | 75.67567567567568 |
| 1 | 65.34181240063593 |
| 4 | 47.535771065182836 |
| 16 | 36.56597774244833 |
### Chart:
| Category | |
|---|---|
| 0.25 | 83.4013605442177 |
| 1 | 89.52380952380952 |
| 4 | 86.66666666666667 |
| 16 | 81.36054421768708 |
### Chart:
| Category | |
|---|---|
| 0.25 | 105.97938144329898 |
| 1 | 85.36082474226805 |
| 4 | 78.55670103092784 |
| 16 | 69.48453608247422 |
### Chart:
| Category | |
|---|---|
| 0.25 | 100.27624309392264 |
| 1 | 86.60220994475138 |
| 4 | 75.13812154696132 |
| 16 | 62.154696132596676 |
### Chart:
| Category | |
|---|---|
| 0.25 | 102.22575516693165 |
| 1 | 95.07154213036567 |
| 4 | 94.91255961844196 |
| 16 | 100.0 |
### Chart:
| Category | |
|---|---|
| 0.25 | 78.02874743326488 |
| 1 | 61.19096509240247 |
| 4 | 36.755646817248454 |
| 16 | 18.069815195071868 |
### Chart:
| Category | |
|---|---|
| 0.25 | 72.76853252647503 |
| 1 | 60.21180030257185 |
| 4 | 59.455370650529495 |
| 16 | 52.49621785173979 |
### Chart:
| Category | |
|---|---|
| 0.25 | 66.53061224489795 |
| 1 | 52.38095238095239 |
| 4 | 46.80272108843538 |
| 16 | 32.51700680272109 |
### Chart:
| Category | |
|---|---|
| 0.25 | 78.06041335453101 |
| 1 | 97.77424483306838 |
| 4 | 94.91255961844199 |
| 16 | 97.45627980922099 |
### Chart:
| Category | |
|---|---|
| 0.25 | 106.83624801271861 |
| 1 | 106.35930047694755 |
| 4 | 100.47694753577106 |
| 16 | 101.58982511923688 |Figure S2: neutralizing activities of anti-Gc mAbs against replication-defective VSV carrying SFTS virus Gn/Gc. x-axis indicates concentration of the mAb used to treat virus and y-axis indicates reporter-positive cell number percentages against control (mAb 0 µg/mL). Experiments were performed at triplicates. Data are the means ± standard deviations.

## Slide 3
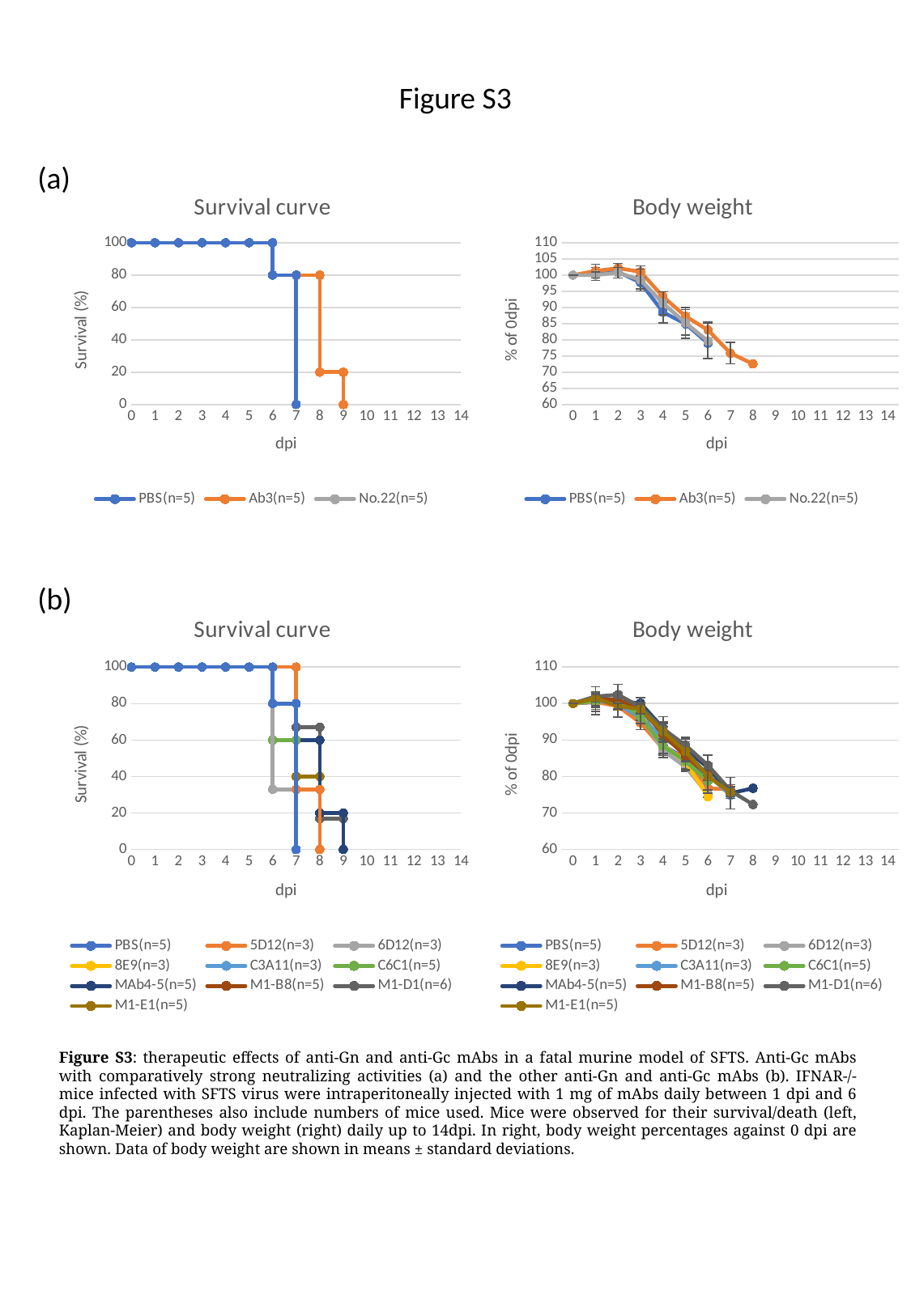

Figure S3
(a)
### Chart:
| Category | PBS(n=5) | Ab3(n=5) | No.22(n=5) |
|---|---|---|---|
### Chart:
| Category | PBS(n=5) | Ab3(n=5) | No.22(n=5) |
|---|---|---|---|
| 0 | 100.0 | 100.0 | 100.0 |
| 1 | 100.36511108122447 | 101.26291887984117 | 100.01476173889967 |
| 2 | 100.96357982410477 | 102.14059587374955 | 100.75237653814231 |
| 3 | 97.70545697835894 | 101.02631310362366 | 98.51390287067282 |
| 4 | 88.54933593754825 | 93.33881563065553 | 91.30448715529724 |
| 5 | 84.95304678754303 | 87.2812824546866 | 85.22176604108753 |
| 6 | 78.92825219188924 | 83.0253255133654 | 79.61445580238458 |
| 7 | None | 75.85906471390248 | None |
| 8 | None | 72.53886010362694 | None |
| 9 | None | None | None |
| 10 | None | None | None |
| 11 | None | None | None |
| 12 | None | None | None |
| 13 | None | None | None |
| 14 | None | None | None |(b)
### Chart:
| Category | PBS(n=5) | 5D12(n=3) | 6D12(n=3) | 8E9(n=3) | C3A11(n=3) | C6C1(n=5) | MAb4-5(n=5) | M1-B8(n=5) | M1-D1(n=6) | M1-E1(n=5) |
|---|---|---|---|---|---|---|---|---|---|---|
### Chart:
| Category | PBS(n=5) | 5D12(n=3) | 6D12(n=3) | 8E9(n=3) | C3A11(n=3) | C6C1(n=5) | MAb4-5(n=5) | M1-B8(n=5) | M1-D1(n=6) | M1-E1(n=5) |
|---|---|---|---|---|---|---|---|---|---|---|
| 0 | 100.0 | 100.0 | 100.0 | 100.0 | 100.0 | 100.0 | 100.0 | 100.0 | 100.0 | 100.0 |
| 1 | 100.36511108122447 | 100.46978580430442 | 100.74728092297255 | 101.11186756464254 | 100.8594371328151 | 100.73735696100323 | 101.84392549184064 | 101.55050744023656 | 101.92815231628295 | 101.43508151895745 |
| 2 | 100.96357982410477 | 99.24089697168385 | 101.17803324830992 | 100.67468805226319 | 99.81333453762664 | 101.14165735788723 | 100.56534604390889 | 100.91215665897 | 102.39549543711492 | 99.28708115804461 |
| 3 | 97.70545697835894 | 94.73065673116349 | 96.97728956939655 | 97.05094803878997 | 96.10248917673194 | 96.42067980079527 | 100.05622160812106 | 98.78906847778154 | 99.07977616185809 | 98.50251313467753 |
| 4 | 88.54933593754825 | 87.61535651824715 | 87.13823216248811 | 88.66692636252469 | 90.64993462256324 | 88.3200761439293 | 93.29848840884745 | 91.55454958110377 | 93.07121482154095 | 92.30218671897475 |
| 5 | 84.95304678754303 | 85.23925212305524 | 82.65118474899515 | 83.97704848509757 | 86.50021128138717 | 84.17535459558172 | 87.58044463540028 | 85.53999086744396 | 88.56338031435679 | 86.90979900150151 |
| 6 | 78.92825219188924 | 76.78503724147858 | 75.0915750915751 | 74.48979591836734 | 80.52191273156409 | 78.74218732372121 | 82.41897376591493 | 80.49460387575814 | 83.08993551442529 | 80.09364990890188 |
| 7 | None | 76.45051194539249 | None | None | 74.86338797814207 | None | 75.43327229992077 | None | 76.14151507709254 | 75.64489795918367 |
| 8 | None | None | None | None | None | None | 76.78571428571429 | None | 72.34848484848486 | None |
| 9 | None | None | None | None | None | None | None | None | None | None |
| 10 | None | None | None | None | None | None | None | None | None | None |
| 11 | None | None | None | None | None | None | None | None | None | None |
| 12 | None | None | None | None | None | None | None | None | None | None |
| 13 | None | None | None | None | None | None | None | None | None | None |
| 14 | None | None | None | None | None | None | None | None | None | None |Figure S3: therapeutic effects of anti-Gn and anti-Gc mAbs in a fatal murine model of SFTS. Anti-Gc mAbs with comparatively strong neutralizing activities (a) and the other anti-Gn and anti-Gc mAbs (b). IFNAR-/- mice infected with SFTS virus were intraperitoneally injected with 1 mg of mAbs daily between 1 dpi and 6 dpi. The parentheses also include numbers of mice used. Mice were observed for their survival/death (left, Kaplan-Meier) and body weight (right) daily up to 14dpi. In right, body weight percentages against 0 dpi are shown. Data of body weight are shown in means ± standard deviations.

## Slide 4
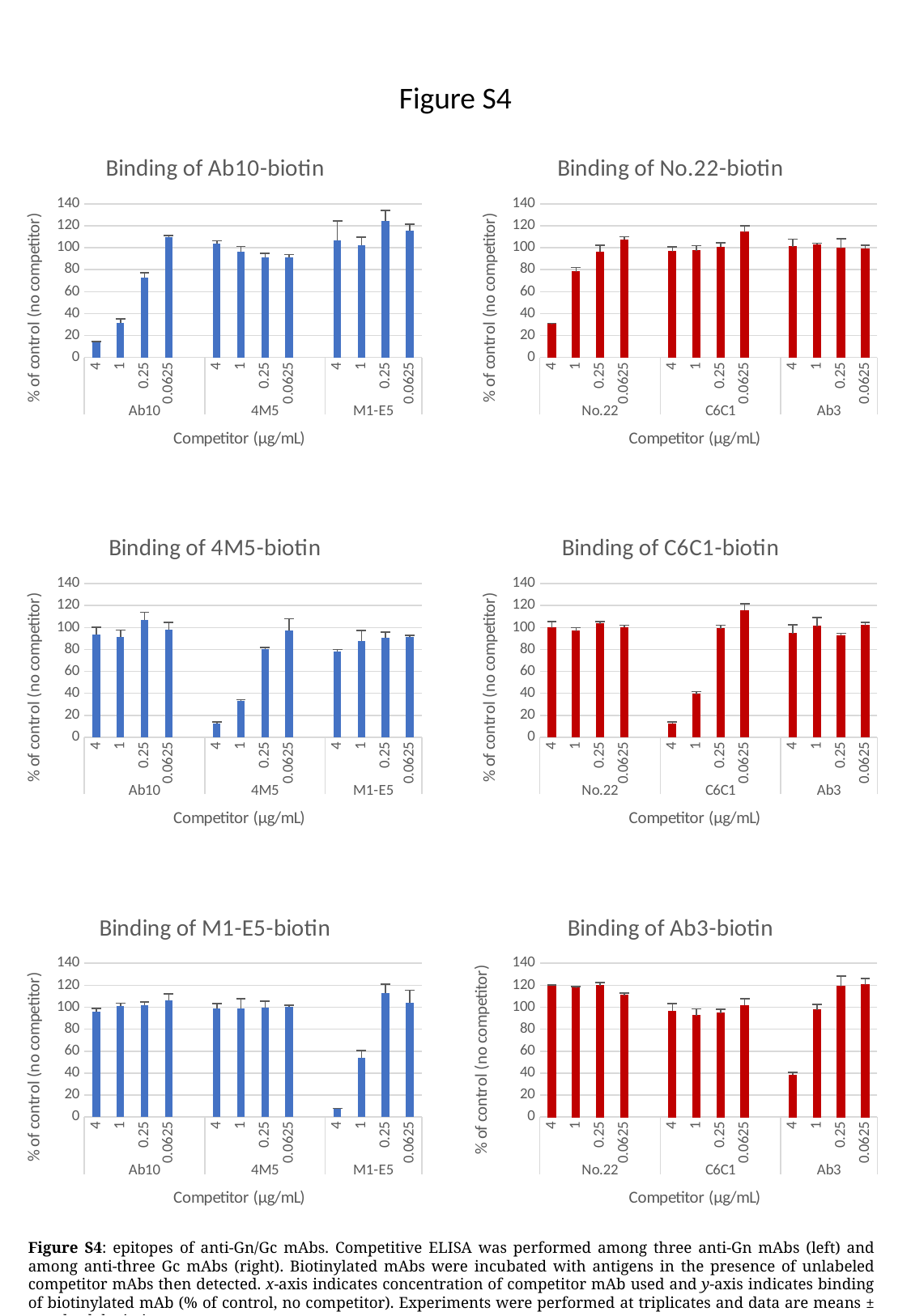

Figure S4
### Chart:
| Category | |
|---|---|
| 4 | 13.968184789714536 |
| 1 | 31.553715406406628 |
| 0.25 | 72.80453257790369 |
| 0.0625 | 109.56635432556114 |
| | None |
| 4 | 103.7853107344633 |
| 1 | 96.7231638418079 |
| 0.25 | 91.29943502824858 |
| 0.0625 | 91.2241054613936 |
| | None |
| 4 | 107.10394421442582 |
| 1 | 102.63674003050774 |
| 0.25 | 124.36260623229464 |
| 0.0625 | 115.86402266288952 |
### Chart:
| Category | |
|---|---|
| 4 | 30.134665876175152 |
| 1 | 78.20784280511562 |
| 0.25 | 96.21411027356653 |
| 0.0625 | 107.19064961463539 |
| | None |
| 4 | 96.54071314529004 |
| 1 | 97.51640943764413 |
| 0.25 | 100.8515167642363 |
| 0.0625 | 114.13872627284017 |
| | None |
| 4 | 101.19068162208801 |
| 1 | 102.69197584124244 |
| 0.25 | 99.65487489214841 |
| 0.0625 | 99.24072476272649 |
### Chart:
| Category | |
|---|---|
| 4 | 93.52066362753158 |
| 1 | 91.21889245945744 |
| 0.25 | 106.6586951647859 |
| 0.0625 | 97.85516777520364 |
| | None |
| 4 | 12.654705236177227 |
| 1 | 33.291834492859756 |
| 0.25 | 80.6737458806298 |
| 0.0625 | 97.32698645184915 |
| | None |
| 4 | 78.37979224273222 |
| 1 | 87.85591510350497 |
| 0.25 | 90.89006800687541 |
| 0.0625 | 91.09931993124577 |
### Chart:
| Category | |
|---|---|
| 4 | 99.82592182307327 |
| 1 | 96.78746637126129 |
| 0.25 | 103.29166007279633 |
| 0.0625 | 100.22155404336131 |
| | None |
| 4 | 12.438677005855359 |
| 1 | 39.16758980851401 |
| 0.25 | 99.05048267130876 |
| 0.0625 | 115.4296565912328 |
| | None |
| 4 | 94.71228615863141 |
| 1 | 101.30637636080873 |
| 0.25 | 92.61275272161743 |
| 0.0625 | 101.89735614307934 |
### Chart:
| Category | |
|---|---|
| 4 | 95.57795600029431 |
| 1 | 101.02273563387534 |
| 0.25 | 101.74380104480906 |
| 0.0625 | 106.4822308880877 |
| | None |
| 4 | 98.56966528715601 |
| 1 | 99.16503303564946 |
| 0.25 | 99.9491759239091 |
| 0.0625 | 100.3412473680389 |
| | None |
| 4 | 7.504966521963063 |
| 1 | 54.00632771687145 |
| 0.25 | 113.06011331027884 |
| 0.0625 | 104.30431903465528 |
### Chart:
| Category | |
|---|---|
| 4 | 119.38082737394768 |
| 1 | 117.31691862043992 |
| 0.25 | 119.83343894269937 |
| 0.0625 | 110.85362541866569 |
| | None |
| 4 | 96.66560408033155 |
| 1 | 92.34300286898313 |
| 0.25 | 94.72744660503668 |
| 0.0625 | 101.63850812878547 |
| | None |
| 4 | 38.325566447051266 |
| 1 | 97.49317049654508 |
| 0.25 | 119.12260967379079 |
| 0.0625 | 120.60099630403344 |Figure S4: epitopes of anti-Gn/Gc mAbs. Competitive ELISA was performed among three anti-Gn mAbs (left) and among anti-three Gc mAbs (right). Biotinylated mAbs were incubated with antigens in the presence of unlabeled competitor mAbs then detected. x-axis indicates concentration of competitor mAb used and y-axis indicates binding of biotinylated mAb (% of control, no competitor). Experiments were performed at triplicates and data are means ± standard deviations.

## Slide 5
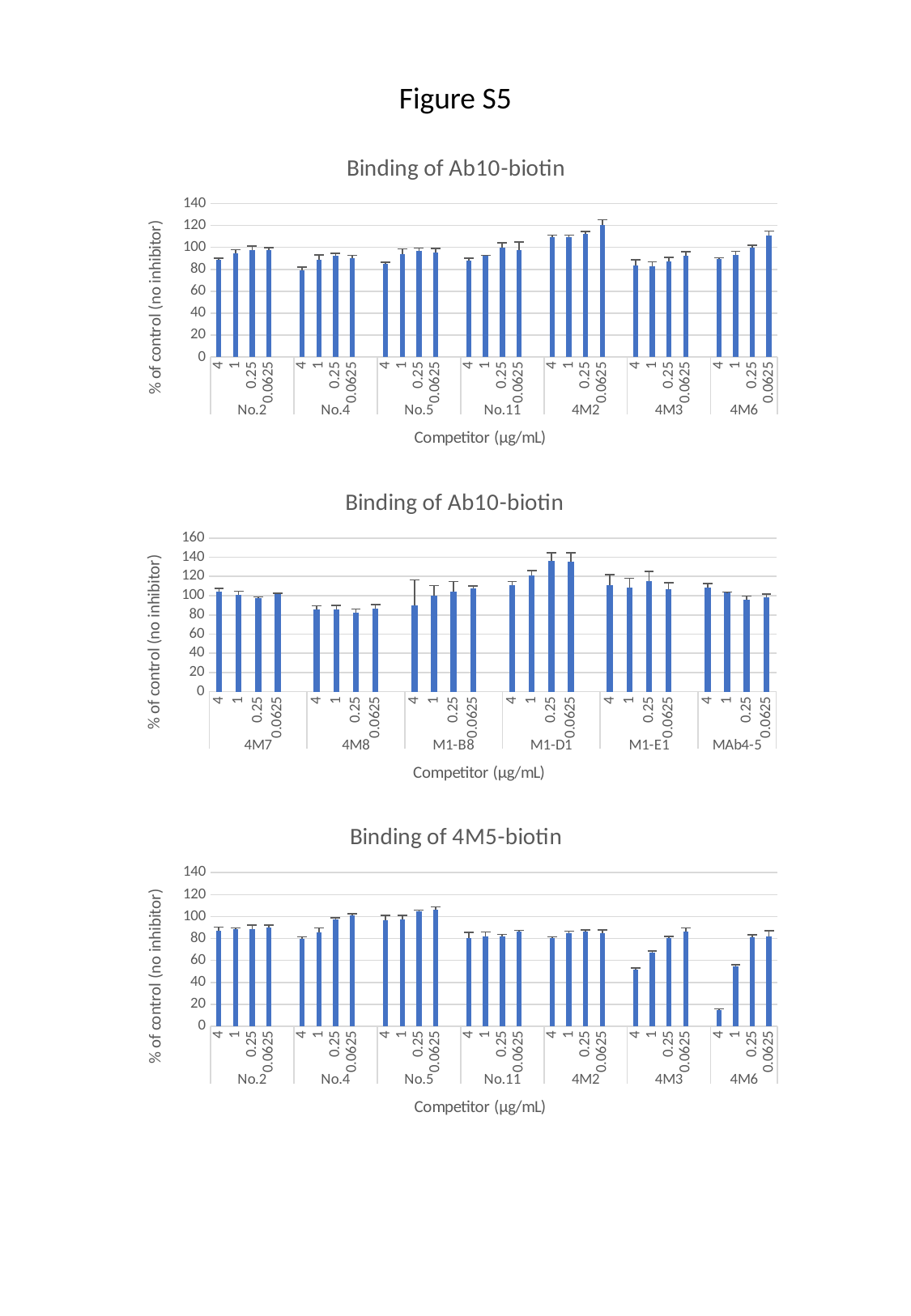

Figure S5
### Chart:
| Category | |
|---|---|
| 4 | 88.58734466504784 |
| 1 | 94.80074275103556 |
| 0.25 | 97.71461219825738 |
| 0.0625 | 97.32895300671332 |
| | None |
| 4 | 79.17440365662047 |
| 1 | 88.98728753035282 |
| 0.25 | 92.35823453792317 |
| 0.0625 | 90.42993858020282 |
| | None |
| 4 | 84.68790172832452 |
| 1 | 94.14369375803456 |
| 0.25 | 96.57191829738606 |
| 0.0625 | 95.50064276531924 |
| | None |
| 4 | 87.61605484930725 |
| 1 | 92.07256106270533 |
| 0.25 | 99.54292243965146 |
| 0.0625 | 97.64319382945293 |
| | None |
| 4 | 109.6700471361234 |
| 1 | 109.45579202971004 |
| 0.25 | 112.26967576060561 |
| 0.0625 | 120.1971146979003 |
| | None |
| 4 | 83.74764595103578 |
| 1 | 82.5235404896422 |
| 0.25 | 87.43879472693033 |
| 0.0625 | 92.58003766478345 |
| | None |
| 4 | 89.74432223968005 |
| 1 | 93.20097128981574 |
| 0.25 | 99.94286530495644 |
| 0.0625 | 110.56991858305956 |
### Chart:
| Category | |
|---|---|
| 4 | 104.14414414414416 |
| 1 | 101.03194103194106 |
| 0.25 | 97.6904176904177 |
| 0.0625 | 101.90008190008193 |
| | None |
| 4 | 86.07698607698609 |
| 1 | 85.37264537264538 |
| 0.25 | 82.11302211302213 |
| 0.0625 | 86.50286650286652 |
| | None |
| 4 | 89.91065591632166 |
| 1 | 99.78208760078451 |
| 0.25 | 104.38003922423188 |
| 0.0625 | 107.53976901285687 |
| | None |
| 4 | 110.93603744149766 |
| 1 | 120.98283931357254 |
| 0.25 | 136.00624024961 |
| 0.0625 | 135.56942277691107 |
| | None |
| 4 | 110.65591632163871 |
| 1 | 108.76007844846372 |
| 0.25 | 114.99237306602747 |
| 0.0625 | 106.40662453693615 |
| | None |
| 4 | 108.26833073322932 |
| 1 | 103.43213728549141 |
| 0.25 | 96.00624024961 |
| 0.0625 | 98.62714508580343 |
### Chart:
| Category | |
|---|---|
| 4 | 87.12638267827725 |
| 1 | 88.5227896760022 |
| 0.25 | 88.53847964226878 |
| 0.0625 | 89.7309170785283 |
| | None |
| 4 | 79.39122930885699 |
| 1 | 85.87118537695146 |
| 0.25 | 97.57590021181454 |
| 0.0625 | 101.23166235192595 |
| | None |
| 4 | 96.90123166235192 |
| 1 | 97.1522711226171 |
| 0.25 | 104.54224523417275 |
| 0.0625 | 105.9386522318977 |
| | None |
| 4 | 80.77194634031538 |
| 1 | 82.12128343924061 |
| 0.25 | 82.15266337177376 |
| 0.0625 | 85.96532517455087 |
| | None |
| 4 | 80.20505309410471 |
| 1 | 84.97986085682899 |
| 0.25 | 86.25411937019406 |
| 0.0625 | 85.06774075430245 |
| | None |
| 4 | 51.439033321127795 |
| 1 | 67.12559502013914 |
| 0.25 | 80.55657268399852 |
| 0.0625 | 86.12229952398388 |
| | None |
| 4 | 14.632002929329916 |
| 1 | 54.52947638227755 |
| 0.25 | 81.1570853167338 |
| 0.0625 | 82.25558403515197 |

## Slide 6
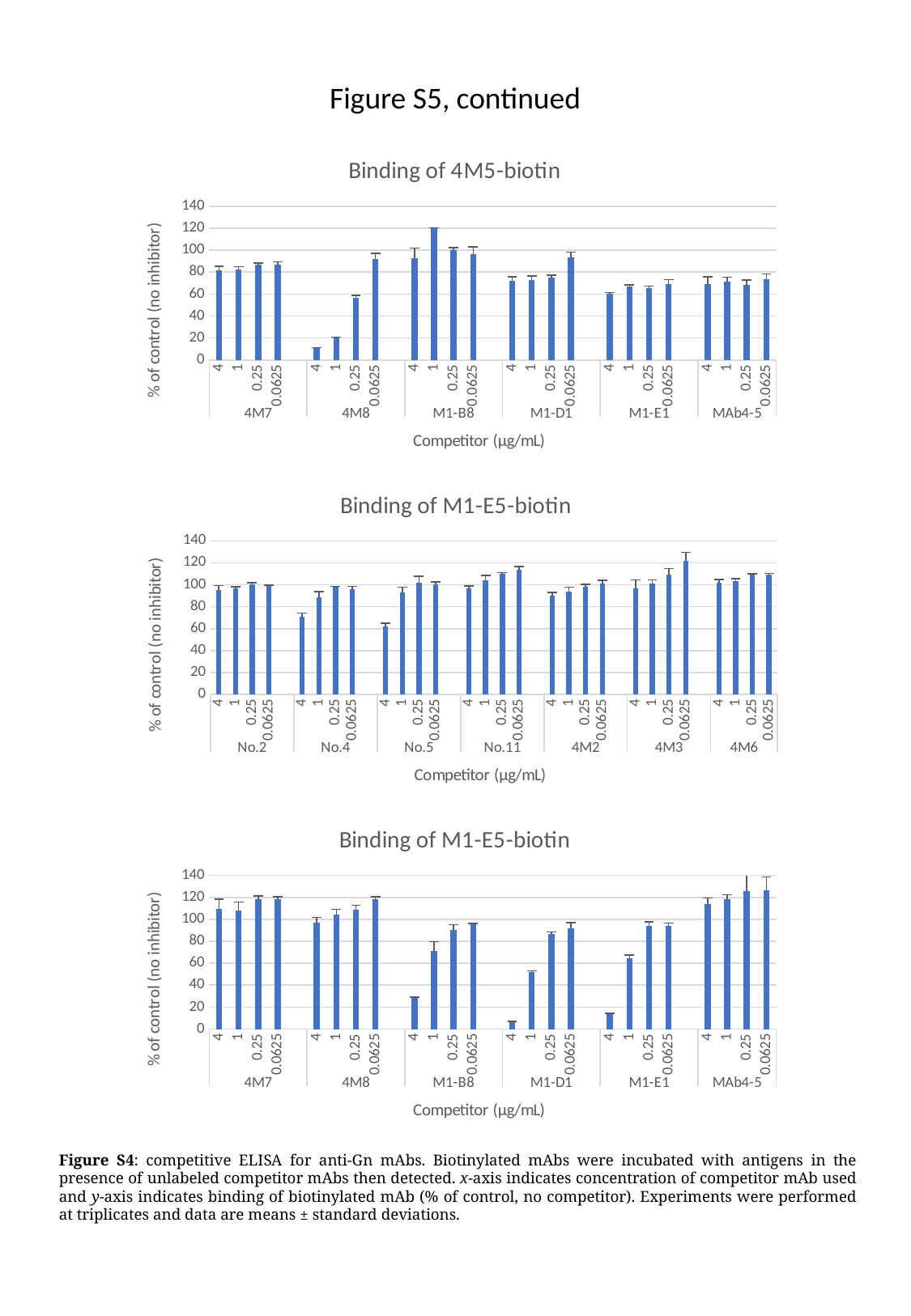

Figure S5, continued
### Chart:
| Category | |
|---|---|
| 4 | 81.43537165873306 |
| 1 | 82.47528377883559 |
| 0.25 | 86.60563896008789 |
| 0.0625 | 87.1329183449286 |
| | None |
| 4 | 10.9352924554959 |
| 1 | 20.090421022887817 |
| 0.25 | 56.83808985589149 |
| 0.0625 | 92.13054535179428 |
| | None |
| 4 | 92.86521616275782 |
| 1 | 120.30234529528111 |
| 0.25 | 100.05651313930487 |
| 0.0625 | 96.43967222379202 |
| | None |
| 4 | 71.77168691720824 |
| 1 | 72.73241028539134 |
| 0.25 | 75.17660356032775 |
| 0.0625 | 93.24667985306581 |
| | None |
| 4 | 59.92078320005978 |
| 1 | 67.11008145878485 |
| 0.25 | 65.76489051640385 |
| 0.0625 | 69.41185262685899 |
| | None |
| 4 | 69.29228009864732 |
| 1 | 71.7136237949331 |
| 0.25 | 68.0965548165309 |
| 0.0625 | 73.76130334055749 |
### Chart:
| Category | |
|---|---|
| 4 | 94.96776354349828 |
| 1 | 96.47492254877335 |
| 0.25 | 100.27631248430043 |
| 0.0625 | 98.85288453487398 |
| | None |
| 4 | 70.7862346144185 |
| 1 | 88.30277149794858 |
| 0.25 | 98.45097546680064 |
| 0.0625 | 95.82182031315415 |
| | None |
| 4 | 62.26241312902956 |
| 1 | 93.0754416813196 |
| 0.25 | 101.74997906723604 |
| 0.0625 | 100.51075944067655 |
| | None |
| 4 | 96.69262329397974 |
| 1 | 104.4293728543917 |
| 0.25 | 110.30729297496441 |
| 0.0625 | 113.80725110943649 |
| | None |
| 4 | 89.91505118710522 |
| 1 | 93.76315980541641 |
| 0.25 | 97.8871705510782 |
| 0.0625 | 101.22703840848037 |
| | None |
| 4 | 96.79310056099807 |
| 1 | 101.3480699991627 |
| 0.25 | 109.41974378296912 |
| 0.0625 | 121.59423930335761 |
| | None |
| 4 | 101.90953314455821 |
| 1 | 103.56494590866187 |
| 0.25 | 109.17011544325855 |
| 0.0625 | 109.30080592463514 |
### Chart:
| Category | |
|---|---|
| 4 | 109.24272126624555 |
| 1 | 108.2262397444275 |
| 0.25 | 118.3329703042184 |
| 0.0625 | 118.02802584767296 |
| | None |
| 4 | 97.11754882741594 |
| 1 | 104.08770783416828 |
| 0.25 | 108.6618746823495 |
| 0.0625 | 118.14419516445219 |
| | None |
| 4 | 28.224560370833633 |
| 1 | 71.4443381649621 |
| 0.25 | 90.39805753807666 |
| 0.0625 | 95.1953498638805 |
| | None |
| 4 | 7.004635420498858 |
| 1 | 52.13744389669633 |
| 0.25 | 86.55728055330731 |
| 0.0625 | 91.94319770436317 |
| | None |
| 4 | 13.891545875947317 |
| 1 | 64.63100581267014 |
| 0.25 | 94.09167831653298 |
| 0.0625 | 94.23883452284599 |
| | None |
| 4 | 113.70071879764758 |
| 1 | 118.12967399985479 |
| 0.25 | 125.39025629855513 |
| 0.0625 | 126.14535685761997 |Figure S4: competitive ELISA for anti-Gn mAbs. Biotinylated mAbs were incubated with antigens in the presence of unlabeled competitor mAbs then detected. x-axis indicates concentration of competitor mAb used and y-axis indicates binding of biotinylated mAb (% of control, no competitor). Experiments were performed at triplicates and data are means ± standard deviations.

## Slide 7
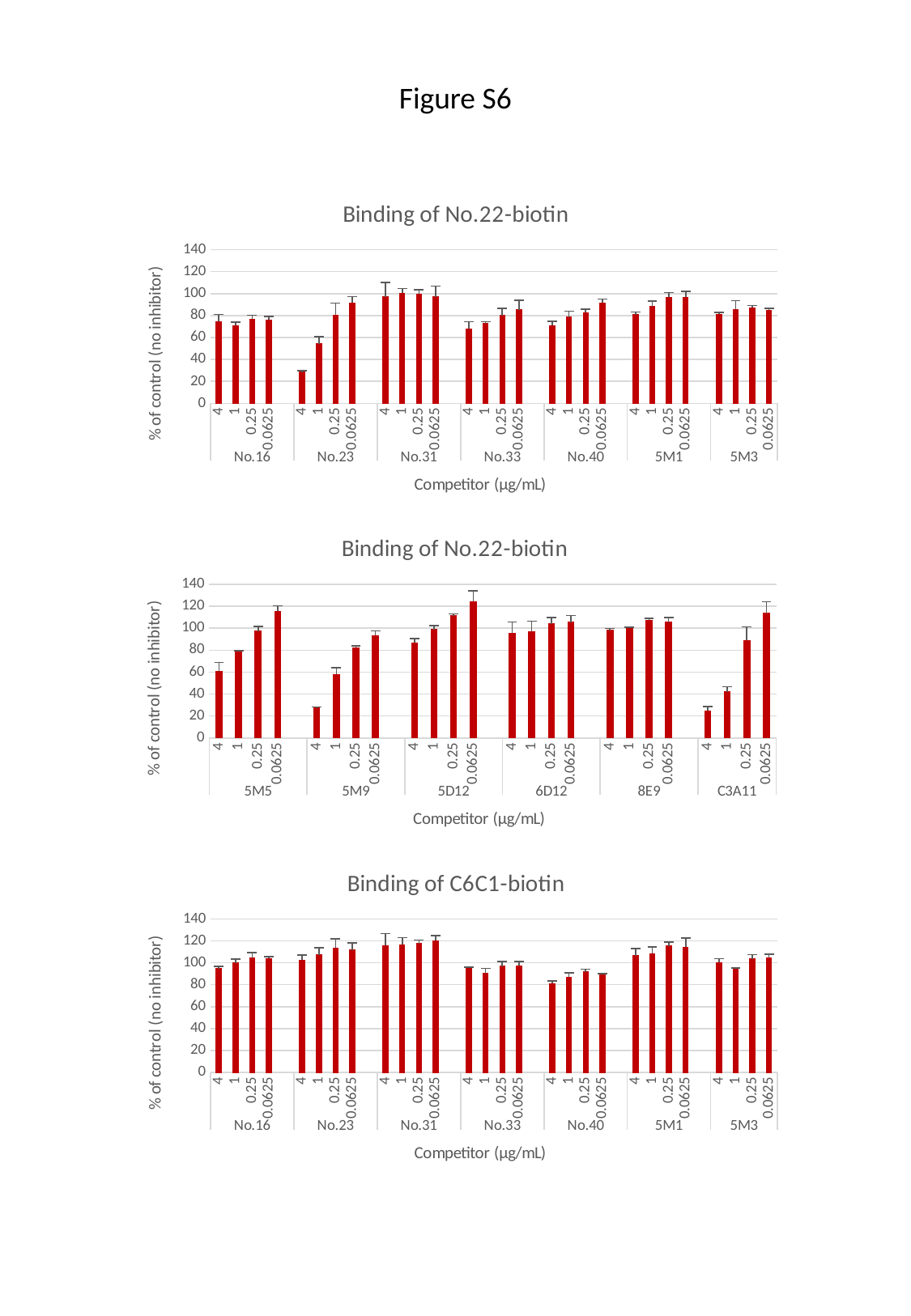

Figure S6
### Chart:
| Category | |
|---|---|
| 4 | 74.55300127713923 |
| 1 | 71.0249042145594 |
| 0.25 | 76.97956577266923 |
| 0.0625 | 76.21328224776502 |
| | None |
| 4 | 28.464240102171136 |
| 1 | 54.18263090676885 |
| 0.25 | 80.36398467432952 |
| 0.0625 | 91.74648786717755 |
| | None |
| 4 | 97.1902937420179 |
| 1 | 100.46296296296299 |
| 0.25 | 99.8403575989783 |
| 0.0625 | 96.93486590038316 |
| | None |
| 4 | 68.16730523627076 |
| 1 | 72.92464878671778 |
| 0.25 | 80.02873563218391 |
| 0.0625 | 85.42464878671778 |
| | None |
| 4 | 70.84929757343554 |
| 1 | 78.75159642401023 |
| 0.25 | 82.66283524904215 |
| 0.0625 | 91.53895274584931 |
| | None |
| 4 | 80.98585584822564 |
| 1 | 88.25273143050735 |
| 0.25 | 96.75616159905142 |
| 0.0625 | 96.79003980689424 |
| | None |
| 4 | 81.3754552384179 |
| 1 | 85.59329211484715 |
| 0.25 | 86.99923774032355 |
| 0.0625 | 84.98348437367663 |
### Chart:
| Category | |
|---|---|
| 4 | 60.54638992371829 |
| 1 | 78.97817988291644 |
| 0.25 | 97.4454497072911 |
| 0.0625 | 115.57566081248892 |
| | None |
| 4 | 27.50910476835776 |
| 1 | 57.84703989158974 |
| 0.25 | 82.40874057762346 |
| 0.0625 | 93.43609723045653 |
| | None |
| 4 | 86.6418307610431 |
| 1 | 98.93560404470463 |
| 0.25 | 111.3358169238957 |
| 0.0625 | 124.26822778073442 |
| | None |
| 4 | 95.65371651587724 |
| 1 | 96.93099166223168 |
| 0.25 | 104.00922476494588 |
| 0.0625 | 105.42841937200637 |
| | None |
| 4 | 98.04860741529181 |
| 1 | 100.37253858435338 |
| 0.25 | 107.34433209153804 |
| 0.0625 | 105.6590384956537 |
| | None |
| 4 | 24.605286499911298 |
| 1 | 42.07912009934362 |
| 0.25 | 88.96576193010465 |
| 0.0625 | 113.712967890722 |
### Chart:
| Category | |
|---|---|
| 4 | 94.61940180408294 |
| 1 | 100.01582528881153 |
| 0.25 | 104.20952682386455 |
| 0.0625 | 103.87719575882261 |
| | None |
| 4 | 102.32631745529356 |
| 1 | 107.3587592973572 |
| 0.25 | 113.40401962335814 |
| 0.0625 | 111.789840164583 |
| | None |
| 4 | 115.85693938914386 |
| 1 | 116.36334863111254 |
| 0.25 | 117.78762462414942 |
| 0.0625 | 120.12976736825446 |
| | None |
| 4 | 95.62020662990427 |
| 1 | 90.46079736325031 |
| 0.25 | 96.8878747543893 |
| 0.0625 | 97.21746846675539 |
| | None |
| 4 | 81.27020346073398 |
| 1 | 87.10147683336503 |
| 0.25 | 91.91861570640805 |
| 0.0625 | 89.34524941370348 |
| | None |
| 4 | 106.42506725747745 |
| 1 | 108.41905364772909 |
| 0.25 | 115.47713245766737 |
| 0.0625 | 114.25858521918026 |
| | None |
| 4 | 100.08239842809151 |
| 1 | 94.35253850541928 |
| 0.25 | 103.593839132915 |
| 0.0625 | 104.67135703872725 |

## Slide 8
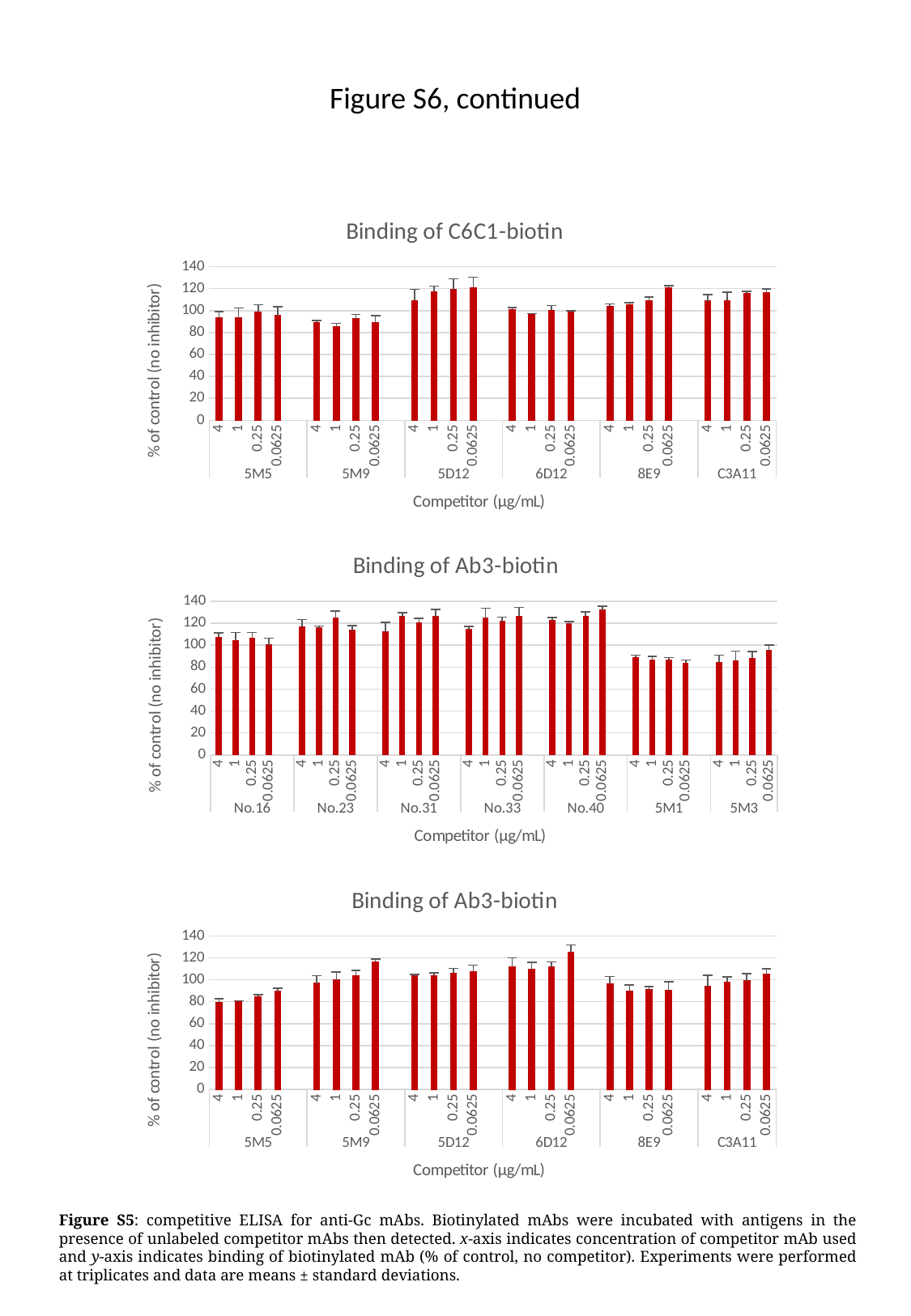

Figure S6, continued
### Chart:
| Category | |
|---|---|
| 4 | 93.55080398824278 |
| 1 | 93.30874301193016 |
| 0.25 | 99.0490461644862 |
| 0.0625 | 95.7062993487407 |
| | None |
| 4 | 89.30897354619331 |
| 1 | 85.7472191804507 |
| 0.25 | 92.72088064088526 |
| 0.0625 | 89.48187424355946 |
| | None |
| 4 | 109.40652549304735 |
| 1 | 117.2771062437517 |
| 0.25 | 119.24020721621376 |
| 0.0625 | 121.13060074525129 |
| | None |
| 4 | 101.31203650884197 |
| 1 | 97.38226532293845 |
| 0.25 | 100.43734550294732 |
| 0.0625 | 99.44856436584901 |
| | None |
| 4 | 104.17158956648184 |
| 1 | 105.42579296555483 |
| 0.25 | 109.22475688448606 |
| 0.0625 | 120.65800236299191 |
| | None |
| 4 | 108.71995850383264 |
| 1 | 109.19255374330011 |
| 0.25 | 115.73972681689817 |
| 0.0625 | 116.62728373004438 |
### Chart:
| Category | |
|---|---|
| 4 | 107.34135964515252 |
| 1 | 104.4265411423916 |
| 0.25 | 106.65339006064994 |
| 0.0625 | 100.46166380012671 |
| | None |
| 4 | 116.7737847379379 |
| 1 | 115.83235267493437 |
| 0.25 | 124.81216619896804 |
| 0.0625 | 113.4425635919254 |
| | None |
| 4 | 112.0847288856703 |
| 1 | 126.133791979723 |
| 0.25 | 120.57572191545215 |
| 0.0625 | 125.98895627772247 |
| | None |
| 4 | 114.40210011767901 |
| 1 | 124.81216619896804 |
| 0.25 | 121.66198968045622 |
| 0.0625 | 126.36914999547388 |
| | None |
| 4 | 122.38616819045895 |
| 1 | 119.43514076219788 |
| 0.25 | 126.55019462297456 |
| 0.0625 | 131.99963791074498 |
| | None |
| 4 | 88.9265885256015 |
| 1 | 86.32017273288095 |
| 0.25 | 86.79827267119062 |
| 0.0625 | 83.76002467612584 |
| | None |
| 4 | 84.3769278223319 |
| 1 | 86.08883405305369 |
| 0.25 | 88.21714990746453 |
| 0.0625 | 95.15731030228255 |
### Chart:
| Category | |
|---|---|
| 4 | 79.47254780999383 |
| 1 | 80.16656384947562 |
| 0.25 | 84.66995681677976 |
| 0.0625 | 89.68229487970389 |
| | None |
| 4 | 96.82294879703886 |
| 1 | 99.96915484268969 |
| 0.25 | 103.93275755706354 |
| 0.0625 | 116.44046884639111 |
| | None |
| 4 | 103.55134179656115 |
| 1 | 104.00128555359152 |
| 0.25 | 106.2992125984252 |
| 0.0625 | 107.63297444962238 |
| | None |
| 4 | 112.10027318013822 |
| 1 | 109.5291659971075 |
| 0.25 | 111.85923188172909 |
| 0.0625 | 125.1165032942311 |
| | None |
| 4 | 96.65330043183219 |
| 1 | 89.9290561381863 |
| 0.25 | 91.28624305983959 |
| 0.0625 | 90.3763109191857 |
| | None |
| 4 | 94.01338858782276 |
| 1 | 98.09372011475932 |
| 0.25 | 99.25406439273193 |
| 0.0625 | 104.96652853044311 |Figure S5: competitive ELISA for anti-Gc mAbs. Biotinylated mAbs were incubated with antigens in the presence of unlabeled competitor mAbs then detected. x-axis indicates concentration of competitor mAb used and y-axis indicates binding of biotinylated mAb (% of control, no competitor). Experiments were performed at triplicates and data are means ± standard deviations.

## Slide 9
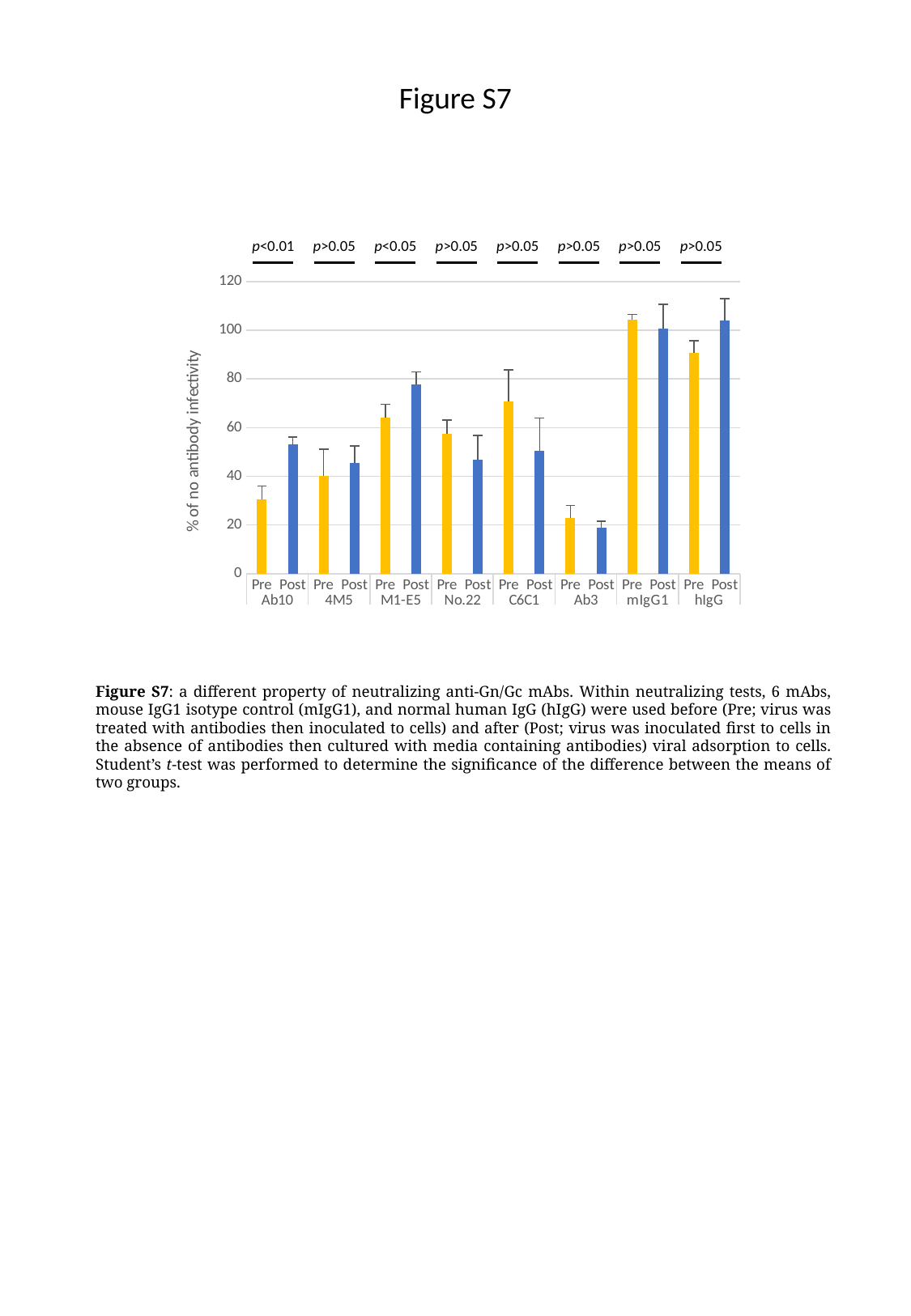

Figure S7
p<0.01
p>0.05
p<0.05
p>0.05
p>0.05
p>0.05
p>0.05
p>0.05
### Chart
| Category | |
|---|---|
| Pre | 30.400000000000002 |
| Post | 53.07692307692307 |
| Pre | 40.0 |
| Post | 45.38461538461539 |
| Pre | 64.0 |
| Post | 77.6923076923077 |
| Pre | 57.6 |
| Post | 46.92307692307693 |
| Pre | 70.80000000000001 |
| Post | 50.38461538461539 |
| Pre | 22.8 |
| Post | 18.846153846153843 |
| Pre | 104.40000000000002 |
| Post | 100.76923076923076 |
| Pre | 90.80000000000001 |
| Post | 103.84615384615385 |Figure S7: a different property of neutralizing anti-Gn/Gc mAbs. Within neutralizing tests, 6 mAbs, mouse IgG1 isotype control (mIgG1), and normal human IgG (hIgG) were used before (Pre; virus was treated with antibodies then inoculated to cells) and after (Post; virus was inoculated first to cells in the absence of antibodies then cultured with media containing antibodies) viral adsorption to cells. Student’s t-test was performed to determine the significance of the difference between the means of two groups.
